# Effect of a Synbiotic Mix on Lymphoid Organs of Broilers Infected with *Salmonella typhimurium* and *Clostridium perfringens*

**DOI:** 10.3390/ani10050886

**Published:** 2020-05-19

**Authors:** Zuamí Villagrán-de la Mora, Olga Vázquez-Paulino, Hugo Avalos, Felipe Ascencio, Karla Nuño, Angélica Villarruel-López

**Affiliations:** 1 Departamento de Ciencias de la Salud, Centro Universitario de Los Altos, Universidad de Guadalajara, Av. Rafael Casillas Aceves 1200, Tepatitlán de Morelos 47620, Jalisco, Mexico; blanca.villagran@academicos.udg.mx; 2 Centro de Investigaciones Biológicas del Noroeste (CIBNOR), Av. Instituto Politécnico Nacional 195, Playa Palo de Santa Rita Sur, La Paz 23096, BCS, Mexico; ascencio@cibnor.mx; 3 Departamento de Farmacobiología, Centro Universitario de Ciencias Exactas e Ingenierías, Universidad de Guadalajara, Blvd. Gral. Marcelino García Barragán 1421, Olímpica, Guadalajara 44430, Jalisco, Mexico; olga.vazquez@academicos.udg.mx (O.V.-P.); avalossanchez@gmail.com (H.A.); 4 Departamento de Ciencias Biomédicas, Centro Universitario de Tonalá, Universidad de Guadalajara, Nuevo Perif. Ote. 555, Ejido San José Tateposco, Tonalá 45425, Jalisco, Mexico

**Keywords:** probiotic, prebiotic, *Clostridium perfringens*, *Salmonella typhimurium*, bursa, spleen, thymus

## Abstract

**Simple Summary:**

The use of synbiotics in the poultry industry could be a tool to regulate immunological activity and generate beneficial effects against pathogens, like *Salmonella typhimurium* and *Clostridium perfringens*, particularly in those cases where the use of antibiotics during poultry production was excluded. Either through the generation of short-chain fatty acids (SCFA) that contribute to mucosa proliferation or promoting the growth of beneficial gut bacteria, synbiotics could favor a microenvironment that improves the activity of the immune system. However, the organization and response of lymphocytes in lymphoid tissues could be modified by the type of active compound of the synbiotic. Therefore, the present work investigated the effect of a synbiotic mix on lymphoid tissues of broilers infected with *Salmonella typhimurium* and *Clostridium perfringens*. The results showed that the mix of probiotics *Lactobacillus rhamnosus* HN001, *Pediococcus acidilactici* MA18/5Ma and a prebiotic can stimulate the bursa and the IgA production, increasing the size of its follicles and promoting the ability to resist infections caused by *S.* *typhimurium* in broilers.

**Abstract:**

Synbiotic consumption can modulate immune response. This work involves studying the effect of a synbiotic on lymphoid organs and IgA of broilers infected with *Salmonella typhimurium* and *Clostridium perfringens*. A total of 258 one-day-old male broilers (*Gallus gallus domesticus*), line COBBAvian48 (free of growth-promoting antibiotics), were distributed into eight treatment groups. A symbiotic mix comprising *Lactobacillus rhamnosus* HN001 and *Pediococcus acidilactici* MA18/5 M as probiotics and 4.5% (0.045 g g^−1^) of *Agave tequilana* fructans as prebiotic *per* dose (one milliliter) was administered through drinking water the first day of life. Bursa, spleen and thymus were analyzed. Broilers treated with the synbiotic, whether or not infected with pathogens, had bigger bursa follicles than the non-treated (*p* < 0.05), and the ones from the synbiotic group had more lymphocytes than the control group (*p* < 0.05). Thymus follicles of the synbiotic group were bigger than the control group (*p* < 0.05). Lesions associated with *Salmonella* infection were found in the bursa, however, in the broilers treated with the synbiotic, the lesions were less intense and were not present after 32 days of life. The synbiotic mix can stimulate the bursa, increasing the size of their follicles and promoting the ability to resist infections caused by *S.* *typhimurium* in broilers.

## 1. Introduction

The poultry industry has been using synthetic antibiotics for immune modulation as feed additives to control pathogens growth [1]. However, because of their effect on promoting antibiotic resistance of microorganisms present in animals [2,3], antibiotics in feces and urine that can spread in the environment [2,4] and in poultry meat [3,5]; and the consumer demand for access to "natural" and antibiotic-free products [6], there is a trend towards decreasing the use of antibiotics as growth promoters [7].

*Salmonella* is a Gram-negative rod-shaped bacteria [8]. The genus *Salmonella* comprises three species, *Salmonella enterica*, *Salmonella bongori* and *Salmonella subterranean*. *S. enterica*, is further classified into six subspecies: *enterica* (subsp. I), *arizonae* (subsp. IIIa), *diarizonae* (subsp. IIIb), *houtenae* (subsp. IV), *indica* (subsp. VI) and *salamae* (subsp. II); with more than 50 serogroups and over 2500 serotypes [9]. *Salmonella enterica* subsp. *enterica* serovar *typhimurium* is the causal agent of white diarrhea [7].

*Clostridium perfringens* is a Gram-positive anaerobic spore-forming bacterium, able to produce various toxins and enzymes responsible for the associated lesions and symptoms. *C. perfringens* strains are classified into five toxinotypes (A, B, C, D and E), based on the production of four major toxins (α, β, ϵ and ι). *C. perfringens* type A causes necrotic enteritis and the subclinical form infection in poultry [4].

Excluding antibiotics as growth promoters during poultry production increases the incidence of certain animal diseases such as those caused by *S. typhimurium* and *C. perfringens* [7]. It has been suggested that synbiotics could offer resistance to gut bacterial infections working as an antibacterial and under normal conditions, probiotics and prebiotics can improve growth performance, intestinal microbial ecology and immunity of poultry [10,11].

The immune system of poultry is a complex, multi-factorial entity. In chickens, comprises primary (bursa of Fabricius and thymus) and secondary (spleen, Harderian gland and mucosa-associated lymphoid tissue [MALT]) immune organs [12]. The immune function is affected by bird age, diet composition, feed and energy intake, genetic potential for growth, environment and stress, among others. This plasticity and responsiveness to external influences have led to many efforts over the years to manipulate immune function through vaccination, reduction or elimination of specific pathogens (e.g., biosecurity), dietary growth promoting antibiotics, nutritional immunomodulation and administration of synbiotics [13].

The intestine—and the microbiota that lives in it—plays an important role in shaping the innate and adaptive immune system [14,15]. It was shown that oral administration of probiotics can significantly affect the systemic and mucosa-associated immune responses, resulting in disease prevention [6]. Probiotics interact with monocytes/macrophages, lymphocytes, dendritic cells and intestinal epithelial cells. Dendritic cells and intestinal epithelial cells can interact and respond to intestinal pathogens through their pattern recognition receptors [16]. Studies with *L. rhamnosus* have shown that this microorganism enhances the expression of TLR9, which was identified as a tumor necrosis factor (TNF) attenuator, showing an inhibitory effect on the reduction of TNF-α induced transepithelial resistance [17].

Poultry production is considered a "stressful system" that acts on birds by generating stress responses with neuroendocrine and metabolic changes [18]. During these responses, some physiological activities like growth, reproduction and digestion may be totally or partially withdraw, directing energy resources to meet other organs’ demands, such as the central nervous system and skeletal muscle. Usually, adrenal hypertrophy coexists with involution of the lymphoid organs, including atrophy of the thymus, bursa, spleen and pancreas, usually larger and faster growing birds are more sensitive to that condition [19].

Studies have tried to modify the immune function of chickens through nutritional compounds like fatty acids and vitamin E, prebiotics, probiotics and synbiotics. However, so far, the effect of synbiotics on the immune system has not been clarified, because while there are studies that indicate that it is possible to modify the immune response through food additives, others report no change at the time of administration [13]. Therefore, the present study was conducted to evaluate the effect of a synbiotic formulated with agave inulin as a prebiotic and *Lactobacillus rhamnosus* and *Pediococcus acidilactici* as probiotics on lymphoid organs and IgA of broilers infected with *Salmonella typhimurium* and *Clostridium perfringens*.

## 2. Materials and Methods

### 2.1. Experimental Birds and Housing

The present study was carried out following the guidelines of the Institutional Animal Care and Use Committee (IACUC) and was approved by the Bioethics Committee (CUCBA) of the University of Guadalajara (Permit Number: CINV.078/15).

Two hundred and fifty-eight one-day-old male broilers (*Gallus gallus domesticus*), line COBBAvian48 (free of growth-promoting antibiotics), obtained from a local commercial hatchery (AVI–INC, Jalisco, Mexico) were housed and distributed randomly into eight treatment groups with three replicates each one. Each replicate was assigned to a pen physically separated from the other two of the same replicates and randomly placed in different sections of the shed. The treatments were: (1) control group (*n* = 43); (2) synbiotic (*n* = 35); (3) synbiotic mix + *S. typhimurium* (*n* = 25); (4) synbiotic mix + *C. perfringens* (*n* = 25); (5) synbiotic mix + *S. typhimurium* + *C. perfringens* (*n* = 25); (6) *S. typhimurium* (*n* = 30); (7) *C. perfringens* (*n* = 30); and (8) *S. typhimurium* + *C. perfringens* (*n* = 45). The experiment was conducted using a randomized complete block design and the blocking variables were the experimental unit (pen of broilers) and the sampling time.

Housing and feeding were performed as Villagran-de la Mora et al [20]. Broilers were vaccinated against avian pox, Gumboro and Newcastle diseases, but they were not vaccinated against *Salmonella* or *Clostridium*.

### 2.2. Synbiotic Mix and Pathogens Administration

A synbiotic mix comprising 7 log of CFU g^−1^ *Lactobacillus rhamnosus* HN001 and *Pediococcus acidilactici* MA18/5 M as probiotics and 4.5% (0.045 g g^−1^) of *Agave tequilana* fructans as prebiotic *per* dose (1 mL) was administered in drinking water the first day of the broilers life. The water containers with the mix were available for 2 h.

For treatment groups 3 to 8, pathogens were administered on day 17 [21]. *S. typhimurium* was subcultured in lactose broth with yeast extract and *C. perfringens* ATCC 13,124 was subcultured in thioglycolate broth and incubated under anaerobic environment; pathogens were separated by centrifugation (thrice at 4000 g for 20 min) and washed in physiological saline solution (0.8% NaCl). The pellets were suspended in physiological saline solution and the number of bacteria in the suspension was calculated using a nephelometer (DensiCHEK, Model: OA009372, bioMérieux, Inc, Missouri, MO, USA). Finally, 5 log CFU of *S. typhimurium* and/or 3 log CFU of *C. perfringens* per bird were administrated through drinking water [22].

To calculate the synbiotic and pathogen intake, we considered that, broilers consume 1.12 mL and 25 mL of water per hour at 1 and 17 days old, respectively [23].

### 2.3. Slaughter and Collection of Samples

Body weight (BW) of each bird was measured weekly. Four sampling times were scheduled with three replicas from each treatment at 22, 32, 36 and 39 days of life. Broilers were euthanized by intraperitoneal injection of 3 mL per 2.5 kg of broiler, 6.3% sodium pentobarbital (PISA Agropecuaria, Guadalajara, Mexico). Cardiac puncture was performed to obtain blood samples [24], which were deposited in collection tubes with coagulation activator applied by spraying (red cap—BD Vacutainer) and centrifuged at 1600× *g* for 10 min to separate the serum, which was aliquoted and stored at −20 °C for later analysis [25]. Subsequently, broilers were eviscerated, and their bursa, spleen and thymus were removed.

### 2.4. Lymphoid Organs Evaluation

Bursa and spleen were weighed, and their morphometric index was calculated according to the following formula [26]:

(1) $$\mathrm{Morphometric}\;\mathrm{index}=\frac{\mathrm{organ}\;\mathrm{weight}\;\left(g\right)}{\mathrm{body}\;\mathrm{weight}\;\left(g\right)}\times 1000$$

Samples of the organs were placed in formaldehyde at 10% (Cat. No. 256462.0905, PanReacAppliChem, Darmstadt, Germany), dehydrated in alcohol (Cat. No. 89370-084, VWR, Radnor, PA, USA) cleared in xylene (Cat. No. 89370-088, VWR, PA) and imbedded in paraffin (Cat. No. P3683, Merck, Darmstadt, Germany). Tissue blocks were cut in a microtome (Cat. No. 4062, SLEE medical GmbH, Mainz, Germany) into 5-mm thick sections and stained with hematoxylin–eosin. Images were analyzed using the software Motic Images Plus 2.0 (Motic, Kowloon, Hong Kong).

Nine complete bursal and thymus follicles, corresponding to three birds per treatment per time, were observed with 4× panoramic objective (Optical microscope model E200 LED, Nikon, Shinagawa, Tokyo, Japan). The follicle section was selected for analysis when the cut passed through the central area of the follicle. Medullar follicular area was determined by a line crossing the basal membrane that divided the two follicular areas. Follicular cortex was determined as total follicular area minus medullar area in each follicle [27]. Lymphocyte counts were performed using the 100X panoramic. Four fields per follicle, two in the cortex and two in the marrow, were counted for the bursa and thymus.

Spleen samples were observed with 100X panoramic (Optical microscope model E200 LED, Nikon) and mature lymphocytes present in the white pulp were counted, reporting all found by field (nine fields per sample). The field was chosen in the area adjacent to some small-caliber blood vessel within the white pulp.

A qualitative analysis was also carried out in the three organs using the objectives 4×, 10× and 40×.

### 2.5. Quantification of IgA

IgA was quantified in serum samples using the ELISA technique (enzyme-linked immunosorbent assay), in duplicates and following the protocol of the Chicken ELISA kit of Abcam IgA (Cat. No. 157691, Abcam, Cambridge, UK); the samples were diluted in 1× diluent (Abcam) and placed in the wells of the plate, incubated in darkness at room temperature for 20 min. Once the incubation time was over, the content of the wells was aspirated, and three rinses were performed with the 1× Wash Buffer (Abcam). After removing the buffer, the enzyme–antibody conjugate 1× (Abcam) was added to each well and incubated in darkness at room temperature for 20 min. At the end of the incubation time, three washes were performed with the 1× Wash Buffer (Abcam) and the TMB substrate solution (Abcam) was added to each well, allowing it to incubate in darkness for 10 min. At the end of the incubation time, the stop solution (Abcam) was added and the absorbance was quantified in a microplate reader at 450 nm (Model 680, Bio-Rad, Hercules, CA, USA).

## 3. Results

The consumption effect of a synbiotic mix on the morphology of the bursa of broilers was evaluated. At 22 days of life, the average morphometric index of the control group birds was 2.5 ± 1.2, however, it was reduced at 39 days, with an average of 1.1 ± 0.3 g. In the synbiotic group, the initial morphometric index was 1.5 ± 0.6 g (22 days of life), increasing until the end of the bioassay (39 days of life) to reach an index of 2.3 ± 0.6 g. It must be noted that although no significant difference was found (*p* > 0.05), this value was higher than the other treatments at the end of the experiment (control 1.1 ± 0.3 g; synbiotic + *S. typhimurium* 0.5 ± 0.3 g; *S. typhimurium* 1.5 ± 1.1 g; synbiotic + *C. perfringens* 1.7 ± 0.3 g; *C. perfringens* 1.1 ± 1.2 g; synbiotic + *S. typhimurium* + *C. perfringens* 1.1 ± 0.5 g; *S. typhimurium* + *C. perfringens* 1.9 ± 0.6) (Table 1).

The morphometric index of the bursa allowed to determine the immunocompetence or immunosuppression in broilers between 7 and 42 days of age. When the index was 1.1 or higher was considered ideal, which translates into an immunocompetent organism. On the contrary, finding indexes equal to or lower than one would result in immunosuppression [28].

**Figure 1:**
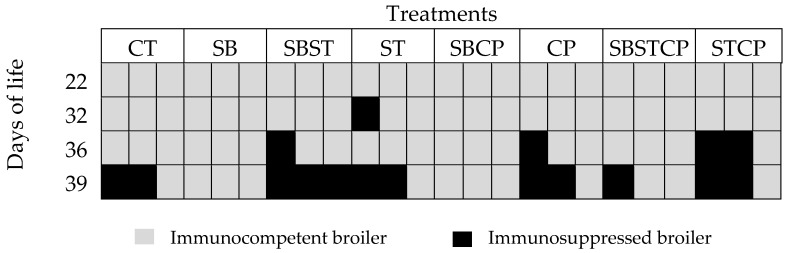
Bursa morphometric index in broilers treated with the synbiotic mix and inoculated with *Salmonella typhimurium* and *Clostridium perfringens*. CT—non-challenged control group; SB—synbiotic; SBST—synbiotic + *S. typhimurium*; ST—*S. typhimurium*; SBCP—synbiotic + *C. perfringens*; CP—*C. perfringens*; SBSTCP—synbiotic + *S. typhimurium* + *C. perfringens*; STCP—*S. typhimurium* + *C. perfringens*. Each square represents one broiler.

There were no broilers with immunosuppression signs in the synbiotic group according to the morphometric index. Meanwhile, immunosuppression was observed in the control group (2 birds) at day 39. At day 36, immunosuppressed broilers were found in the groups synbiotic + *S. typhimurium* (1 bird), *C. perfringens* (1 bird) and *S. typhimurium* + *C. perfringens* (2 birds). At day 39, birds with immunosuppression were found in most groups, except for the synbiotic and *S. typhimurium* + *C. perfringens* groups (Figure 1).

Histological analysis showed that the follicles of the synbiotic group were bigger (*p* < 0.05) in comparison to the control group at 32 (363 ± 111 vs. 161 ± 16 μm) and 39 (304 ± 78 vs. 180 ± 32 μm) days of life. Likewise, the cortex of the synbiotic group had more volume in comparison to the control group (*p* < 0.05) at 32 (196 ± 78 vs. 80 ± 16 μm), 36 (236 ± 67 vs. 98 ± 29 μm) and 39 (174 ± 73 vs. 97 ± 22 μm) days of life. Meanwhile, the medulla of the synbiotic group had more volume (*p* < 0.05) than the control group at 32 (167 ± 78 vs. 82 ± 23 μm) and 36 (134 ± 61 vs. 78 ± 22 μm) days of life (Supplementary Table S1). Larger follicles and cortex are indicative of better immunocompetence in broilers.

For broilers inoculated with *S. typhimurium*, those treated with the synbiotic (synbiotic + *S. typhimurium*) showed larger follicles when compare to those not treated at 32 (351 ± 54 vs. 203 ± 63 μm) and 36 days of life (420 ± 38 vs. 348 ± 41 μm) (Supplementary Table S1).

In the lymphocyte counts *per* follicle of the bursa, it was observed that the follicles of the synbiotic group had a greater number of lymphocytes than the control (*p* < 0.05) at 22 (109 ± 14 vs. 86 ± 31) and 39 days (148 ± 26 vs. 77 ± 15). For broilers inoculated with pathogens, the *S. typhimurium* group showed higher number of lymphocytes than the synbiotic + *S. typhimurium* group at day 22 (105 ± 18 vs. 80 ± 9) (*p* < 0.05); and in the groups inoculated with *C. perfringens*, those who were treated with the synbiotic (synbiotic + *C. perfringens*) had higher lymphocyte counts than those not treated (*C. perfringens*) at day 32 (180 ± 24 vs. 91 ± 24), 36 (148 ± 23 vs. 73 ± 24) and day 39(154 ± 20 vs. 96 ± 23). In broilers treated with both pathogens, the synbiotic group + *S. typhimurium* + *C. perfringens* had more lymphocytes than *S. typhimurium* + *C. perfringens* (*p* < 0.05) at day 32 (154 ± 16 vs. 126 ± 24) (Table 2).

**Figure 2:**
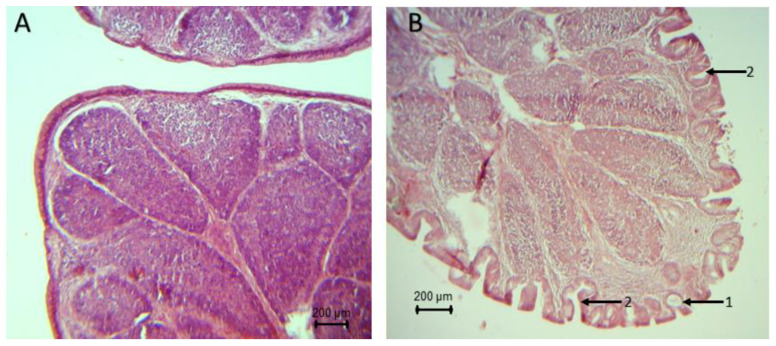
Histopathological lesions in the bursa of broilers challenged with *S. typhimurium* and *C. perfringens.* (**A**): Bursa without apparent lesions (control group) (4×). (**B**): (1) intraepithelial mucosal cysts, (2) epithelial invaginations (S. *typhimurium* + C. perfringens) (4×).

The histopathological analysis of the bursa showed signs of atrophy since day 22 of life in broilers’ groups inoculated with the pathogens (*S. typhimurium* and *S. typhimurium* + *C. perfringens*). Intraepithelial and intrafolytic mucosal cysts, epithelial invaginations, epithelial folds in the folia, atrophy at the apex of the folds, and smaller lymphoid follicles were found. Broilers of the group synbiotic + *S. typhimurium* did not show histopathological lesions after 32 days (Figure 2). It should be noted that no apparent lesions were found on the bursa of broilers in the control, synbiotic, *C. perfringens* and synbiotic + *C. perfringens* groups.

The spleen weight was similar between treatments; at 22 days of life the average weight of the spleens was 1 ± 0.1 g, with a constant increase, until reaching an average weight of 2.3 ± 0.4 g in all treatments at the end of the bioassay (39 days of life). The spleen of the broilers of the *C. perfringens* group was heavier (*p* < 0.05) at 36 days of life (2.76 ± 1.43 vs. 1.60 ± 0.10 g), when compare to the synbiotic + *C. perfringens* group (Table 3).

The spleen morphometric index of the *C. perfringens* group showed a higher rate in comparison to the synbiotic + *C. perfringens* group at 36 days of life (1.5 ± 1 vs. 08 ± 0.1). No other differences were found between treatments (Table 4).

The white pulp lymphocyte count of the spleen was performed and at day 39, the number of lymphocytes of the synbiotic group (148 ± 26) were higher (*p* < 0.05) than the control (77 ± 15). Regarding the birds inoculated with *Salmonella*, those treated with the synbiotic (synbiotic + S. *typhimurium*) showed lower numbers of lymphocytes when compared with the untreated (S. *typhimurium*) at 22 (3 ± 2 vs. 17 ± 7) and 39 (576 vs. 7622) days; contrary to what happened with the broilers inoculated with *C. perfringens*, where those treated with the synbiotic (synbiotic *+ C. perfringens*) had a higher number of lymphocytes at day 39 (154 ± 20) compared to the untreated (*C. perfringens*) group (96 ± 23) (Table 5).

There were no differences in the number of lymphocytes between the synbiotic + *S. typhimurium* + *C. perfringens* and *S. typhimurium* + *C. perfringens* groups (Table 5).

**Figure 3:**
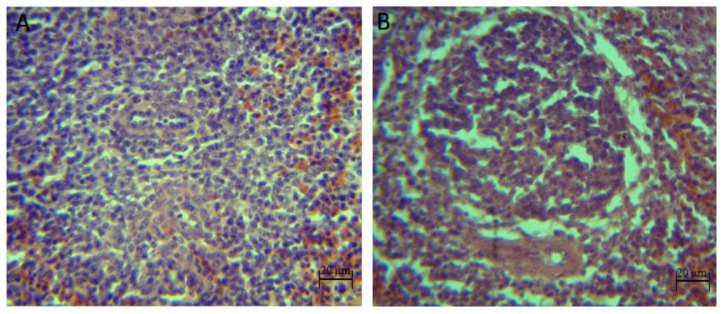
Histopathological injuries in the spleen of broilers chickens challenged with *S. typhimurium* and *C. perfringens***.** (**A**): Spleen without apparent lesions (control group) (40×). (**B**): Lymphoid depletion in the spleen (*S. typhimurium* + *C. perfringens* group) (40×).

The qualitative histopathological analysis showed discrete to moderate lymphoid depletion in the spleen of broilers inoculated with pathogens (*S. typhimurium*, *C. perfringens* and *S. typhimurium* + *C. perfringens* groups) from 22 days of age until the end of the bioassay. However, with the synbiotic and the pathogens (synbiotic mix + *S. typhimurium*, synbiotic mix + *C. perfringens* and synbiotic mix + *S. typhimurium* + *C. perfringens*), no signs of lymphoid depletion at day 39 were shown, also, no histopathological injuries were identified in the control and synbiotic groups (Figure 3).

Histological analysis of thymus showed that the follicles of the control group were smaller (*p* < 0.05) when compared to the synbiotic group at 22 (489 ± 151 vs. 803 ± 251 μm), 36 (474 ± 165 vs. 995 ± 359 μm) and 39 (410 ± 149 vs. 987 ± 195 μm) days of age.

At 22 days of life, broilers of *C. perfringens* group had bigger follicles (1323 ± 331 μm) in comparison to synbiotic + *C. perfringens* group (918 ± 348 μm) (*p* < 0.05). Thymus follicles of the birds from the *S. typhimurium* group were smaller (*p* < 0.05) at day 36 (554 ± 270 μm) than the synbiotic + *S. typhimurium* group (1023 ± 402 μm). The cortex of the thymus follicles of the synbiotic group had more volume when compare to the control group (*p* < 0.05) at 22 (497 ± 173 vs. 292 ± 86 μm), 36 (610 ± 238 vs. 290 ± 138 μm) and 39 (565 ± 195 vs. 248 ± 107 μm) days of life.

At 22 days of life, broilers of the *C. perfringens* group had cortexes with more volume (796 ± 234 μm) compare to the synbiotic + *C. perfringens* group (532 ± 199 μm) (*p* < 0.05). Thymus follicles cortexes of the birds of the *S. typhimurium* group were smaller (*p* < 0.05) at day 36 (343 ± 135 μm) when compare to the synbiotic + *S. typhimurium* group (670 ± 213 μm). The evaluation of the thymus medulla showed that it had a higher volume in the synbiotic group when compared to control the group at 36 (385 ± 236 vs. 183 ± 69 μm) and 39 (423 ± 128 vs. 163 ± 65 μm) days. The same situation was observed in the *C. perfringens* group, who had bigger medulla (*p* < 0.05) when compared to the synbiotic + *C. perfringens* group at 22 (527 ± 165 vs. 387 ± 176 μm) and 32 (628 ± 361 vs. 417 ± 173 μm) days of life (Supplementary Table S2).

Concerning the number of lymphocytes per thymus follicle, the control group had fewer lymphocytes than the synbiotic group (*p* < 0.05) at 22 (37 ± 14 vs. 135 ± 50) and 32 days (83 ± 13 vs. 239 ± 78). In broilers challenged with *C. perfringens* those who were treated with the synbiotic (synbiotic + *C. perfringens* group) had a higher number of lymphocytes at 36 (348 ± 28 vs. 284 ± 52) and 39 days (390 ± 43 vs. 284 ± 53) when compare to those not treated (*C. perfringens*). In broilers challenged with both pathogens, those who were treated with the synbiotic (synbiotic mix + *S. typhimurium* + *C. perfringens)* had a higher number of lymphocytes at day 22 compared to those not treated (298 ± 43 vs. 193 ± 41). However, this was modified at the end of the bioassay (39 days of life), where the broilers of the *C. perfringens* group showed higher counts of lymphocytes than those of the synbiotic + *C. perfringens* group (337 ± 38 vs. 256 ± 43) (Table 6).

**Figure 4:**
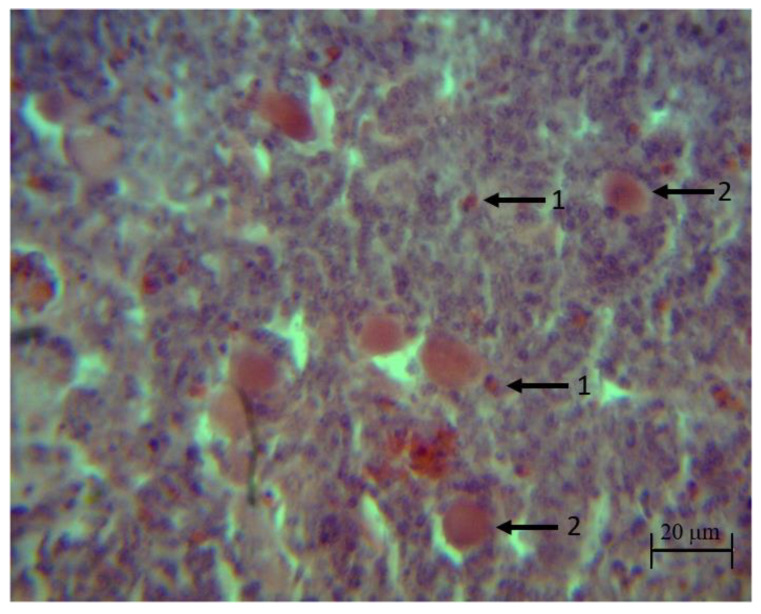
Histopathological injuries in the thymus of broilers challenged with *S. typhimurium* and *C. perfringens* (100×). (1) heterophiles (2) reticular epithelial cells (*S. typhimurium* + *C. perfringens)*

In the histopathological analysis, no apparent injuries were found on the thymus of the control and synbiotic groups during the bioassay. Groups challenged with the pathogens (*S. typhimurium*, *C. perfringens* and *S. typhimurium* + *C. perfringens*) showed discrete to moderate infiltration of heterophils, degeneration and necrosis of reticular epithelial cells in the medulla at 22, 32, 36 and 39 days of life. The groups treated with the synbiotic (synbiotic + *S. typhimurium*, synbiotic + *C. perfringens* and synbiotic + *S. typhimurium* + *C. perfringens*) showed those injuries at 22 and 32 days of life (Figure 4).

The IgA concentration of the control group was higher than the synbiotic group at day 22 (78.7 ± 3.2 vs. 67.5 ± 0.6) (*p* < 0.05), however, this situation changed at day 36 when the synbiotic group showed a higher IgA concentration (*p* < 0.05) when compared to the control (164.4 ± 1.5 vs. 151.7 ± 2.3). In groups infected with *S. typhimurium*, the IgA concentration was significantly higher in the synbiotic + *S. typhimurium* group in all samples (*p* < 0.05); similar results were found in the chickens infected with *C. perfringens*, where the IgA concentration of the synbiotic + *C. perfringens* group was higher than those of the *C. perfringens* group in most samples (*p* < 0.05). A higher IgA concentration was found in the synbiotic + *S. typhimurium* + *C. perfringens* group when compared to its control, at all times except the last sampling (day 39) (146.4 ± 1.1 vs. 84.9 ± 2.6) (*p* < 0.05) (Table 7).

## 4. Discussion

Bursa, thymus and spleen are the producers of immune cells, orientation of the cells occurs more efficiently in healthy animals than in immune-compromised ones, higher relative weights of lymphoid organs could be the consequence of amplified B and T lymphocytes. In disease-free animals, an increase in weight of immune organs correlates with enhanced proliferation of immune cells, which represents better immunity of the body [29].

In assessing the bursa weight, the synbiotic group was identified as the only group that showed sustained bursa growth throughout the bioassay, the variation of the weight of the bursa in the other groups may be the result of stress caused by pathogenic microorganisms inoculated to birds. According to the anatomic-physiological development of the bursa of Fabricius in normal conditions and free of stress, it is expected that this organ maintains a sustained growth until broilers reach eight weeks of life, where it will naturally begin the process of atrophy. Several authors have reported moderate atrophy of the bursa towards the sixth or seventh week in birds subjected to different challenges such as bedding reuse, Gumboro disease infection [30,31], immobilization, turning [19], heat stress [32] and overcrowding [33]. Decreased bursa weight is known to be associated with immunosuppression, Gomes et al., [33] found that by decreasing bursa weight, broilers are more susceptible to enteritis caused by *Salmonella enterica subsp. enterica serovar* Enteritidis.

In the spleen weight of the broilers treated with the synbiotic no differences were found. Similar results were reported by Alkhalf et al. (2010), who found no difference in the spleen weight of broilers treated with a probiotic. This may indicate that the spleen develops its proper functions with age [34]. However, it has been reported an increase in the size and weight of chickens’ spleens treated with probiotics and synbiotics [35,36].

Although the morphometric index of the bursa of broilers inoculated with the pathogenic microorganisms (*S. typhimurium*, *C. perfringens* and *S. typhimurium* + *C. perfringens*) did not indicate immunosuppression, the histopathological analysis of the organs revealed signs of atrophy at day 22; changes in the integrity as well as in the function of the bursa can cause changes in the production of immunoglobulins [33] which implies immunosuppression. These results indicate that macroscopic evaluation of the bursa does not always reflect the immune capacity or damage degree of the bursa [30]. It should be noted that no signs of atrophy were found in the histopathological analysis of the synbiotic group, which is consistent with the morphometric index (equal to or greater than 1.1) found in that group.

The development of lymphoid tissues can reflect the immune system response and functionality. Bursa of Fabricius, spleen and thymus are the main immune organs involved in humoral and cell immunity of animals [37]. Bursa of Fabricius is required for B-cell development, the function of spleen, the biggest peripheral immune organ, is involved in the chicken’s immune reaction, and thymus is the site of T-cell maturation [38,39].

In this study, the follicles from the bursa of broilers treated with the synbiotic, inoculated or not with pathogens, showed a significant cortex increase (*p* < 0.05) when compared to those who were not treated. When evaluating the thymus, this tendency only occurred between the synbiotic and control groups, where the cortex of the synbiotic group’s follicles was thicker (*p* < 0.05) than those of the control group. The histomorphological variations in the cortex and medulla of bursa and thymus, have a link with immune function. A larger cortex could be a sign of the rapid maturation rate of thymocytes [29] which can be proven by noting that those follicles with a larger cortex also had a higher lymphocyte count when compared to those with a smaller cortex, also it has been reported that specific probiotics like *B. infantis*, *B. animalis* [40], *L. acidophilus*, *L. reuteri*, *L. salivarius* [41] and *L. rhamnosus* [42] have been shown to induce an increase in lymphocytes B and T.

The spleen evaluation showed a lymphoid depletion in broilers challenged with *S. typhimurium* and *C. perfringens*, as published by Parsons et al., this depletion is part of the histopathological changes caused by *S. typhimurium* [43].

The histopathological lesions found in the bursa of the challenged broilers are associated to *Salmonella* infection. Loss of lymphoid tissue and degeneration of the bursa, has been reported by Garcia et al., Kumari et al. and Lopes et al., who suggested that the lesions of the bursa could have resulted from adverse physiological conditions related with *Salmonella* infection, like anorexia, dehydration, anemia, etc. [44,45,46]. It should be noted that in broilers challenged with *S. typhimurium*, but treated with the synbiotic, the lesions occurred only at 22 and 32 days of life and showed less intensity when compared to untreated broilers.

No apparent lesions were found in the bursa of chickens challenged with *C. perfringens* in this work, although the presence of *C. perfringens* is associated with an increased risk of infectious bursal disease [47]; similar results were reported by Ao et al [48].

In general, broilers treated with the synbiotic, challenged or not with the pathogens, had higher IgA concentration than untreated ones. It was reported that IgA production can be induced by the administration of probiotics. Sakai et al. [49], found that oral administration of *Lactobacillus gasseri* LG2055 induced IgA production and increased the rate of IgA+ cell population in Peyer’s patch and in the lamina propria. Similar results were reported by Gao et al., when administered *L. plantarum* strain IMAU10120 (LP-8) [50]

IgA plays an important role in host defense against mucosal transmitted pathogens, prevents commensal bacteria from binding to epithelial cells and neutralizes their toxins to maintain homeostasis at the mucosal surfaces, also, secretion of IgA is critical in the regulation of the composition of the microbial community in the gut [40]

## 5. Conclusions

The synbiotic composed of *Lactobacillus rhamnosus* HN001, *Pediococcus acidilactici* MA18/5 M and *Agave tequilana* fructans can stimulate the bursa and the IgA production, increasing the size of its follicles and promoting the ability to resist infections caused by *S. typhimurium* in broilers.

## Figures and Tables

**Table 1:** Bursa morphometric index of broilers treated with the synbiotic mix and inoculated with *Salmonella typhimurium* and *Clostridium perfringens*.

Days of Life	Treatments							
	CT	SB	SBST	ST	SBCP	CP	SBSTCP	STCP
22	2.5 ± 1.2	1.5 ± 0.6	2.8 ± 0.6	1.6 ± 0.7	1.9 ± 0.5	2.6 ± 0.2	1.6 ± 0.6	1.3 ± 0.1
32	2.3 ± 0.2	2 ± 0.3	1.9 ± 0.5	4.1 ± 3.6	2 ± 1	4 ± 2	2.8 ± 1	5.9 ± 2.9
36	2 ± 0.4	2.4 ± 0.6	1.4 ± 0.5	1.5 ± 0.5	1.4 ± 0.1	1.4 ± 1	3.5 ± 2.4	1.6 ± 1.5
39	1.1 ± 0.3	2.3 ± 0.6	0.5 ± 0.3	1.5 ± 1.1	1.7 ± 0.3	1.1 ± 1.2	1.1 ± 0.5	1.9 ± 0.6

Data represent means from three replicates per treatment; CT—non-challenged control group; SB—synbiotic; SBST—synbiotic + *S. typhimurium*; ST—*S. typhimurium*; SBCP—synbiotic + *C. perfringens*; CP—*C. perfringens*; SBSTCP—synbiotic + *S. typhimurium* + *C. perfringens*; STCP—*S. typhimurium* + *C. perfringens*.

**Table 2:** Synbiotic effect on the number of lymphocytes per bursa follicle in broilers challenged with *Salmonella typhimurium* and *Clostridium perfringens*.

Days of Life	Lymphocytes							
	CT	SB	SBST	ST	SBCP	CP	SBST CP	STCP
22	86 ± 31 ^a^	109 ± 14 ^b^	80 ± 9 ^A^	105 ± 18 ^B^	96 ± 14	80 ± 22	87 ± 10 ^A^	125 ± 32 ^B^
32	128 ± 27	138 ± 30	91 ± 21	92 ± 20	180 ± 24 ^a^	91 ± 24 ^b^	154 ± 16 ^A^	126 ± 24 ^B^
36	135 ± 38 ^a^	103 ± 13 ^b^	78 ± 13	85 ± 22	148 ± 23 ^a^	73 ± 24 ^b^	140 ± 12	152 ± 27
39	77 ± 15 ^a^	148 ± 26 ^b^	57 ± 6	76 ± 22	154 ± 20 ^a^	96 ± 23 ^b^	99 ± 26	87 ± 21

CT: control group; SB: synbiotic mix; SBST: synbiotic mix + *S. typhimurium*; ST: *S. typhimurium*; SBCP: synbiotic mix + *C. perfringens*; CP: *C. perfringens*; SBSTCP: synbiotic mix + *S. typhimurium* + *C. perfringens*; STCP: *S. typhimurium* + *C. perfringens*. ^a,b,A,B^ Values with different superscripts in the row are significantly different between adjacent columns (*p* < 0.05).

**Table 3:** Effect of dietary supplementation with a synbiotic mix on spleen weight of broilers orally inoculated with *Salmonella typhimurium* and *Clostridium perfringens*.

Days of Life	Spleen Weight							
	CT	SB	SBST	ST	SBCP	CP	SBSTCP	STCP
22	0.9 ± 0.1	0.9 ± 0.1	1 ± 0.1	1 ± 0.6	1 ± 0.1	1 ± 0.1	1 ± 0	1 ± 0.1
32	1.3 ± 0.6	1.3 ± 0.6	0.7 ± 0.3	1.3 ± 0.6	1.3 ± 0.6	1.3 ± 0.6	2 ± 0	1.3 ± 0.6
36	1.4 ± 0.2	2.3 ± 0.3	1.8 ± 0.6	1.1 ± 0.3	1.6 ± 0.1 ^a^	2.8 ± 1.4 ^b^	1.1 ± 0.4	1.5 ± 0.2
39	1.8 ± 1	1.8 ± 0.3	2.2 ± 0.6	2.2 ± 0.3	2.5 ± 0	2.3 ± 0.6	2.5 ± 0.5	2.7 ± 0.3

CT—non-challenged control group; SB—synbiotic; SBST—synbiotic + *S. typhimurium*; ST—*S. typhimurium*; SBCP—synbiotic + *C. perfringens*; CP—*C. perfringens*; SBSTCP—synbiotic + *S. typhimurium* + *C. perfringens*; STCP—*S. typhimurium* + *C. perfringens.* ^a,b^ Values with different superscripts in the row are significantly different (*p* < 0.05).

**Table 4:** Spleen morphometric index of broilers treated with the synbiotic mix and inoculated with *Salmonella typhimurium* and *Clostridium perfringens*.

Days of Life	Treatments							
	CT	SB	SBST	ST	SBCP	CP	SBSTCP	STCP
22	1.2 ± 0.1	1 ± 0.1	1.3 ± 0.3	1.1 ± 0	1.1 ± 0	1.2 ± 0	1.2 ± 0.1	1.3 ± 0.2
32	1.1 ± 0.4	1.1 ± 0.4	0.5 ± 0.2	1.8 ± 0.8	1 ± 0.4	1.1 ± 0.5	1.8 ± 0.2	1.3 ± 0.9
36	0.8 ± 0.1	1.3 ± 0	1.1 ± 0.3	0.9 ± 0.2	0.8 ± 0.1 ^a^	1.5 ± 1 ^b^	0.6 ± 0.1	0.8 ± 0.1
39	0.8 ± 0.5	0.7 ± 0.1	0.8 ± 0.2	0.8 ± 0	85 ± 22	0.8 ± 0.3	1.1 ± 0.2	1 ± 0.1

Data represent means from three replicates per treatment. CT—non-challenged control group; SB—synbiotic; SBST—synbiotic + *S. typhimurium*; ST—*S. typhimurium*; SBCP—synbiotic + *C. perfringens*; CP—*C. perfringens*; SBSTCP—synbiotic + *S. typhimurium* + *C. perfringens*; STCP—*S. typhimurium* + *C. perfringens.* ^a,b^ Means with different superscripts within the same row differ significantly.

**Table 5:** Synbiotic effect on the white pulp lymphocyte count of broilers’ spleens challenged with *Salmonella typhimurium* and *Clostridium perfringens*.

Days of Life	Lymphocytes							
	CT	SB	SBST	ST	SBCP	CP	SBSTCP	STCP
22	8 ± 3	7 ± 9	3 ± 2 ^A^	17 ± 7 ^B^	9 ± 4	9 ± 7	12 ± 9	9 ± 3
32	3 ± 1	3 ± 2	6 ± 4	10 ± 4	11 ± 5	14 ± 8	17 ± 10	15 ± 7
36	5 ± 5	5 ± 5	5 ± 4	9 ± 4	12 ± 5	17 ± 5	8 ± 6	7 ± 3
39	77 ± 15 ^a^	148 ± 26 ^b^	57 ± 6 ^A^	76 ± 22 ^B^	154 ± 20 ^a^	96 ± 23 ^b^	99 ± 26	87 ± 21

CT: control group; SB: synbiotic mix; SBST: synbiotic mix + *S. typhimurium*; ST: *S. typhimurium*; SBCP: synbiotic mix + *C. perfringens*; CP: *C. perfringens*; SBSTCP: synbiotic mix + *S. typhimurium* + *C. perfringens*; STCP: *S. typhimurium* + *C. perfringens*. ^a,b,A,B^ Values with different superscripts in the row are significantly different between adjacent columns (*p* < 0.05).

**Table 6:** Synbiotic effect on the count of lymphocytes *per* thymus follicle in broilers challenged with *Salmonella typhimurium* and *Clostridium perfringens* in different days of life.

Days of Life	Lymphocytes							
	CT	SB	SBST	ST	SBCP	CP	SBSTCP	STCP
22	37 ± 14 ^A^	135 ± 50 ^B^	147 ± 52 ^a^	93 ± 28 ^b^	150 ± 34	172 ± 40	298 ± 43 ^a^	193 ± 41 ^b^
32	83 ± 13 ^A^	239 ± 78 ^B^	204 ± 17	204 ± 46	278 ± 36	287 ± 50	275 ± 23	270 ± 49
36	155 ± 32	151 ± 22	200 ± 30	167 ± 29	348 ± 28 ^A^	284 ± 52 ^B^	260 ± 58	263 ± 47
39	126 ± 24	150 ± 21	186 ± 35	213 ± 49	390 ± 43 ^A^	284 ± 53 ^B^	256 ± 43 ^a^	337 ± 38 ^b^

CT: control group; SB: synbiotic mix; SBST: synbiotic mix + *S. typhimurium*; ST: *S. typhimurium*; SBCP: synbiotic mix + *C. perfringens*; CP: *C. perfringens*; SBSTCP: synbiotic mix + *S. typhimurium* + *C. perfringens*; STCP: *S. typhimurium* + *C. perfringens*. ^a,b,A,B^ Values with different superscripts in the row are significantly different between adjacent columns (*p* < 0.05).

**Table 7:** Synbiotic effect on serum IgA concentration in broilers challenged with *Salmonella typhimurium* and *Clostridium perfringens* (ng mL^−1^) in different days of life.

Treatments	ng mL^−1^			
	22 days	32 days	36 days	39 days
CT	78.7 ± 3.2	117 ± 1.8 ^a^	151.7 ± 2.3	156 ± 2.8 ^a^
SB	67.5 ± 0.6	112.4 ± 3.9 ^a^	164.4 ± 1.5	156.5 ± 1.7 ^a^
SBST	79.5 ± 2.3	222.1 ± 1.5	196.9 ± 1.9	166.2 ± 1.6
ST	59 ± 0.2	146.4 ± 2.3	171.2 ± 1.3	77.2 ± 3.6
SBCP	45.2 ± 0.7	182.8 ± 1.1	119.1 ± 0.1 ^a^	101.9 ± 1.4
CP	37 ± 0.4	169.3 ± 2.9	125.6 ± 1.7 ^a^	87.67 ± 5.4
SBSTCP	83 ± 2.6	149.2 ± 0.7	217.87 ± 1.5	84.9 ± 2.6
STCP	66.5 ± 2.6	161 ± 1.1	205.8 ± 6	146.4 ± 1.1

CT: control group; SB: synbiotic mix; SBST: synbiotic mix + *S. typhimurium*; ST: *S. typhimurium*; SBCP: synbiotic mix + *C. perfringens*; CP: *C. perfringens*; SBSTCP: synbiotic mix + *S. typhimurium* + *C. perfringens*; STCP: *S. typhimurium* + *C. perfringens*. ^a^ Means with superscripts in the same column are homogeneous (*p* < 0.05).

## Supplementary Materials

The following are available online at https://www.mdpi.com/2076-2615/10/5/886/s1, Table S1: Morphology of the bursa of broilers treated with the synbiotic mix and inoculated with *Salmonella typhimurium* and *Clostridium perfringens*, Table S2: Morphology of the thymus of broilers treated with the synbiotic mix and inoculated with *Salmonella typhimurium* and *Clostridium perfringens*.
