# Supplementary material for: Effect of a Synbiotic Mix on Lymphoid Organs of Broilers Infected with Salmonella typhimurium and Clostridium perfringens
Source: Animals (Basel). 2020 May 19;10(5):886. doi: 10.3390/ani10050886 (PMC7278420; doi:10.3390/ani10050886)
Supplement: Supplementary file 1 [file animals-10-00886-s001.zip › TABLE S1 Compartments of the bursa.docx]

**Table 1S.** Morphology of the bursa of broilers treated with the synbiotic mix and inoculated with *Salmonella* Typhimurium and *Clostridium perfringens*.

| **PARAMETERS (μm)** | **TREATMENTS** | | | | | | | |
| --- | --- | --- | --- | --- | --- | --- | --- | --- |
|  | CT | SB | SBST | ST | SBCP | CP | SBSTCP | STCP |
| 22 days of life  Follicle  Cortex  Medulla | 281±67  114±38  167±47 | 304±55  147±36  157±45 | 314±71  172±73  142±31 | 340±46  173±49  152±51 | 340±46  112±27  228±61 | 342±75  130±27  211±74 | 275±65  99±16^A^  176±61 | 311±63  146±34^B^  165±63 |
| 32 days of life  Follicle  Cortex  Medulla | 161±16^a^  80±16^a^  82±23^a^ | 363±111^b^  196±78^b^  167±78^b^ | 351±54 ^A^  203±70  148±29 | 203±63^B^  70±28^B^  133±50 | 344±68  188±44^a^  156±49^a^ | 312±60  95±32^b^  217±68^b^ | 454±107^A^  278±102^A^  176±50 | 337±55^B^  116±30^B^  221±60 |
| 36 days of life  Follicle  Cortex  Medulla | 176±31^a^  98±29^a^  78±22^a^ | 370±95^b^  236±67^b^  134±61^b^ | 420±38^A^  279±47^A^  142±50 | 348±41^B^  161±48^B^  186±58 | 347±62  189±71  158±45 | 342±68  160±44  182±55 | 380±63  193±56  187±52 | 376±61  130±55  246±79 |
| 39 days of life  Follicle  Cortex  Medulla | 180±32^a^  97±22^a^  83±22 | 304±78^b^  174±73^b^  130±40 | 334±102  201±69^A^  133±55 | 301±87  132±37^B^  170±81 | 415±109^a^  195±52^a^  220±84 | 288±60^b^  81±33^b^  207±53 | 360±110  181±79  179±97 | 315±55  137±49  178±60 |

Means with different superscripts within the same row differ significantly

*Data represent means from three replicates per treatment.

CT – non-challenged control group; SB – Synbiotic; SBST – Synbiotic + *S.* Typhimurium; ST – *S.* Typhimurium; SBCP – Synbiotic + *C. perfringens;* CP – *C. perfringens;* SBSTCP *–* Synbiotic + *S.* Typhimurium + *C. perfringens;* STCP - *S.* Typhimurium + *C. perfringens*

Values with different superscripts in the row (a - b or A - B) are significantly different between adjacent columns (p<0.05).

| **Compar-timentos** (μm) | **Tratamientos** | | | | | | | |
| --- | --- | --- | --- | --- | --- | --- | --- | --- |
|  | CT | SB | SBST | ST | SBCP | CP | SBSTCP | STCP |
| 18 días  Folículo  Corteza  Médula | 317±142  127±51  189±108 | 295±92  129±40  166±57 | 302±73  150±44  152±57 | 312±24  157±26  155±26 | 317±67  110±26^a^  206±63 | 327±45  175±39^b^  151±32 | 305±68  140±42^A^  165±44 | 245±90  59±20^B^  186±77 |
| 22 días  Folículo  Corteza  Médula | 281±67  114±38  167±47 | 304±55  147±36  157±45 | 314±71  172±73  142±31 | 340±46  173±49  152±51 | 340±46  112±27  228±61 | 342±75  130±27  211±74 | 275±65  99±16^A^  176±61 | 311±63  146±34^B^  165±63 |
| 25 días  Folículo  Corteza  Médula | 135±18^a^  66±14^a^  69±13^a^ | 320±74^b^  158±45^b^  162±75^b^ | 351±82  190±88^A^  161±52 | 310±62  111±19^B^  198±75 | 280±50  104±34  176±51^a^ | 263±40  144±31  119±43^b^ | 373±61  141±27  232±56 | 367±73  113±47  254±73 |
| 32 días  Folículo  Corteza  Médula | 161±16^a^  80±16^a^  82±23^a^ | 363±111^b^  196±78^b^  167±78^b^ | 351±54 ^A^  203±70  148±29 | 203±63^B^  70±28^B^  133±50 | 344±68  188±44^a^  156±49^a^ | 312±60  95±32^b^  217±68^b^ | 454±107^A^  278±102^A^  176±50 | 337±55^B^  116±30^B^  221±60 |
| 36 días  Folículo  Corteza  Médula | 176±31^a^  98±29^a^  78±22^a^ | 370±95^b^  236±67^b^  134±61^b^ | 420±38^A^  279±47^A^  142±50 | 348±41^B^  161±48^B^  186±58 | 347±62  189±71  158±45 | 342±68  160±44  182±55 | 380±63  193±56  187±52 | 376±61  130±55  246±79 |
| 39 días  Folículo  Corteza  Médula | 180±32^a^  97±22^a^  83±22 | 304±78^b^  174±73^b^  130±40 | 334±102  201±69^A^  133±55 | 301±87  132±37^B^  170±81 | 415±109^a^  195±52^a^  220±84 | 288±60^b^  81±33^b^  207±53 | 360±110  181±79  179±97 | 315±55  137±49  178±60 |
