# Supplementary material for: Effect of a Synbiotic Mix on Lymphoid Organs of Broilers Infected with Salmonella typhimurium and Clostridium perfringens
Source: Animals (Basel). 2020 May 19;10(5):886. doi: 10.3390/ani10050886 (PMC7278420; doi:10.3390/ani10050886)
Supplement: Supplementary file 1 [file animals-10-00886-s001.zip › TABLE S2 comartments of the thymo.docx]

**Table 2S.** Morphology of the thymus of broilers treated with the synbiotic mix and inoculated with *Salmonella* Typhimurium and *Clostridium perfringens*.

| PARAMETERS (μm) | TREATMENTS | | | | | | | |
| --- | --- | --- | --- | --- | --- | --- | --- | --- |
|  | CT | SB | SBST | ST | SBCP | CP | SBSTCP | STCP |
| 22 days of life  Follicle  Cortex  Medulla | 489±151^a^  292±86 ^a^  107±75 | 803±251^b^  497±173^b^  305±114 | 1103±531  678±337  425±218 | 1209±188  730±135  479±111 | 918±348^a^  532±199^a^  387±176^a^ | 1323±331^b^  796±234^b^  527±165^b^ | 784±240  511±185  273±133 | 811±166  506±104  305±106 |
| 32 days of life  Follicle  Cortex  Medulla | 372±151^a^  197±73  175±87 | 856±368^b^  590±267  266±137 | 1166±327  742±240  425±168 | 1115±423  678±298  437±240 | 974±286  556±122  417±173^a^ | 1398±525  770±215  628±361^b^ | 905±388  555±155  350±250 | 1526±2297  1221±2247  305±123 |
| 36 days of life  Follicle  Cortex  Medulla | 474±165^a^  290±138^a^  183±69 | 995±359^b^  610±238^b^  385±236 | 1023±402^A^  670±213^A^  353±250^A^ | 554±270^B^  343±135^B^  211±146^B^ | 1004±616  660±472  344±209 | 844±334  508±206  336±183 | 695±241  440±188  522±99 | 1013±470  625±279  388±209 |
| 39 days of life  Follicle  Cortex  Medulla | 410±149^a^  248±107^a^  163±65^a^ | 987±195^b^  565±195^b^  423±128^b^ | 1166±485  690±269  476±237 | 956±376  621±178  335±244 | 887±290  580±197  308±188 | 1029±232  670±202  360±119 | 641±213  447±166  194±82 | 634±190  403±138  230±73 |

*Data represent means from three replicates (i.e. pens) per treatment.

CT – non-challenged control group; SB – Synbiotic; SBST – Synbiotic + *S.* Typhimurium; ST – *S.* Typhimurium; SBCP – Synbiotic + *C. perfringens;* CP – *C. perfringens;* SBSTCP *–* Synbiotic + *S.* Typhimurium + *C. perfringens;* STCP - *S.* Typhimurium + *C. perfringens*

Values with different superscripts in the row (a - b or A - B) are significantly different between adjacent columns (p<0.05).
